# Case of Coccyodynia

**Published:** 1870-02

**Authors:** W. R. Fox

**Affiliations:** San Leandro, Cal.


ARTICLE VII.

### CASE OF COCCYODYNIA.

By W. R. FOX, M.D., San Leandro, Cal.

Mrs. K., aged 23, a resident of Wilmington, Ill., consulted me February 1st, 1869, about a severe pain in the region of the coccyx. I learned from her, that ten months previous to this date she was delivered of her first child, after a tedious labor. Her recovery from the accouchment was imperfect, suffering for months from symptoms of anæmia.

Although her general health had improved somewhat, under tonic and restorative treatment, yet she complained greatly of pain in the lower part of the back. The pain was very much aggravated by walking, sitting down, or rising from the sitting posture. Upon examination, I found the lower joint of the coccyx to be motionless and tender to the touch. The pelvic organs were in a healthy condition, except that there was slight prolapsus uteri. There was no vaginismus. I pronounced the case to be coccyodynia, and advised amputation of a portion of the coccyx. As she had obtained no relief from treatment, but was growing worse, she readily consented to the operation.

The operation was performed in the following manner:—The patient was placed on the right side; and with the index-finger in the rectum, I made firm pressure outwardly. Then an incision was made on the median line, down to the bone, and of sufficient length to admit of disarticulation at the second joint. The two lower bones were then separated from their attachments and severed at the second joint with small bone forceps. The wound was closed with metallic sutures, and the greater part healed by the first intention. My friend, Dr. E. R. Willard, assisted at the operation.

The result, in this case, was perfectly satisfactory. In a few weeks, Mrs. K. was attending to her household duties, free from pain, having greatly improved mentally and physically. A short time since, I received a note from her saying she was in the enjoyment of good health, the operation having been a success, etc.

Dr. J. C. Nott, formerly of Mobile, recommended and performed this operation 25 years ago, 15 years before the attention of the profession was called to it by Professor Simpson.

